# MoS_2_/MXene Aerogel with Conformal Heterogeneous Interfaces Tailored by Atomic Layer Deposition for Tunable Microwave Absorption

**DOI:** 10.1002/advs.202101988

**Published:** 2022-01-23

**Authors:** Junjie Yang, Jianqiao Wang, Huiqin Li, Ze Wu, Youqiang Xing, Yunfei Chen, Lei Liu

**Affiliations:** ^1^ School of Mechanical Engineering Southeast University Nanjing 211189 P. R. China

**Keywords:** aerogels, atomic layer deposition, heterogeneous interfaces, microwave absorption, MoS_2_ films

## Abstract

In the design of electromagnetic (EM) wave absorbing materials, it is still a great challenge to optimize the relationship between the attenuation capability and impedance matching synergistically. Herein, a 3D porous MoS_2_/MXene hybrid aerogel architecture with conformal heterogeneous interface has been built by atomic layer deposition (ALD) based on specific porous templates to optimize the microwave absorption (MA) performance comprehensively. The original porous structure of pristine Ti_3_C_2_T*_x_* aerogel used as templates can be preserved well during ALD fabrication, which prolongs the reflection and scattering path and ameliorates the dielectric loss. Meanwhile, plenty of heterointerfaces between MoS_2_ and Ti_3_C_2_T*_x_* have been fabricated based on conformally ALD‐deposited MoS_2_ with controlled thickness on the porous surfaces of the templates, which can effectively optimize the impedance matching and transform its response to EM waves from shielding into absorbing. Moreover, the interaction between the attenuation capability and impedance matching can also be modulated by the number of ALD cycle in MoS_2_ fabrication. After optimization, MoS_2_/MXene hybrid aerogel obtained under 300 ALD cycles shows a minimum reflection loss of −61.65 dB at the thickness of 4.53 mm. In addition, its preferable lightweight, high surface area, mechanical, and hydrophobicity properties will also be conducive to further practical applications.

## Graphical abstract

MoS_2_/MXene aerogels with plenty of heterointerface are fabricated based on conformally depositing MoS_2_ onto the surface of Ti_3_C_2_T*_x_* aerogel by atomic layer deposition. The conformal heterointerface between MoS_2_ and Ti_3_C_2_T*_x_* can optimize the impedance matching. The integration of the microstructure and absorption materials can prolong the reflection and scattering path of electromagnetic waves and enhance the attenuation capability.

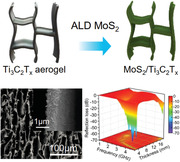

## 1 Introduction

Electromagnetic (EM) wave absorbing materials can extremely absorb and thus attenuate incident EM waves, which is an available way to solve the increasingly serious EM problems in the military and civilian fields.^[^ ^1^, ^2^, ^3^, ^4^ ^]^ Generally, microwave absorption (MA) materials minimize the reflection of EM waves by converting EM energy into other forms as much as possible.^[^ ^5^, ^6^, ^7^, ^8^, ^9^, ^10^ ^]^ Among the family of EM waves absorbing materials, 2D materials are regarded as the most potential candidates, such as graphene,^[^ ^11^, ^12^, ^13^, ^14^ ^]^ MoS_2_,^[^ ^15^, ^16^, ^17^, ^18^ ^]^ and MXene,^[^ ^19^, ^20^, ^21^ ^]^ which also possess the advantages of large absorption bandwidth, excellent reflection loss, and lightweight.

Especially since the breakthrough discovery of 2D Ti_3_C_2_T*_x_* in the excellent electromagnetic interference (EMI) shielding was first reported in 2016,^[^ ^22^ ^]^ the theory, experiment, and application of MXene in these related fields have been extensively studied.^[^ ^23^, ^24^, ^25^, ^26^, ^27^, ^28^, ^29^ ^]^ In particular, MXene containing Ti element usually possesses the ability to block and absorb EM waves,^[^ ^20^, ^22^, ^23^, ^30^, ^31^ ^]^ and its performance is even better than that of the metal films commonly used in most electrical equipment.^[^ ^23^ ^]^ The high performance of MXene is contributed by its excellent electrical conductivity,^[^ ^32^, ^33^, ^34^ ^]^ large specific surface area,^[^ ^35^, ^36^, ^37^ ^]^ light weight,^[^ ^1^, ^38^ ^]^ rich surface functional groups, and surface defects.^[^ ^39^, ^40^, ^41^ ^]^ However, pristine MXene or MXene film will produce a high interface reflection and poor impedance matching,^[^ ^42^, ^43^, ^44^, ^45^, ^46^ ^]^ so that the incident EM waves will be completely reflected rather than absorbed, which is extremely adverse to improve the MA performance.

In order to eliminate the above‐mentioned disadvantages, an effective strategy involved coating pristine Ti_3_C_2_T*_x_* aerogel templates with conformal MoS_2_ by atomic layer deposition (ALD)^[^ ^47^, ^48^, ^49^, ^50^ ^]^ has been proposed to yield numerous heterogeneous interfaces between MoS_2_ and Ti_3_C_2_T*_x_*. Moreover, assembling 2D Ti_3_C_2_T*_x_* into a 3D porous aerogel structure can avoid the aggregation of MXene sheets, thus greatly reduces material density and increase specific surface area,^[^ ^51^, ^52^ ^]^ which will conduce to providing more interfaces and tortuous space to elongate the path of EM waves and adjust the impedance matching. Besides, some multifunctional attributes which aerogel itself possesses will also facilitate its potential applications in various environments.^[^ ^53^ ^]^ The conformal MoS_2_ coating on the surface of 3D Ti_3_C_2_T*_x_* aerogel templates can modulate the EM parameters and optimize the impedance matching, thus promoting the absorbing properties.

Therefore, in this work hybrid aerogels with conformal heterointerfaces architecture are employed to achieve higher MA properties. The pristine Ti_3_C_2_T*_x_* aerogel templates have been prepared 2D based on MXene sheets via a multistep procedure combined with ice‐template, thawing process, and freeze‐drying. The as‐prepared aerogels via directional freeze‐drying possess a better long‐range oriented microstructure compared with those prepared by ordinary freeze‐drying,^[^ ^54^, ^55^ ^]^ which can intensify their absorption capability due to inner multiple reflections and scatterings. Then, the conformal heterointerfaces between MoS_2_ and Ti_3_C_2_T*_x_* are fabricated in a controlled ALD, by which the absorbing properties of MoS_2_/MXene aerogel can be effectively modulated and remarkably enhanced.

## 2 Results and Discussion

**Figure 1:**
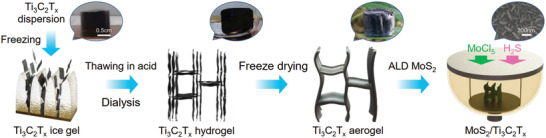
Schematic illustration of the preparation process of MoS_2_/MXene hybrid aerogel.

The fabrication of MoS_2_/MXene hybrid aerogels is illustrated in **Figure** **1**, and the detailed parameters of these samples are included in the Experimental Section, and all sample labels are listed in Table S1, Supporting Information. ALD‐made MoS_2_ nanoflakes can be observed on the surface of Ti_3_C_2_T*_x_* aerogel, identified by Raman characteristic peaks (E^1^_2g_ and A_1g_ modes of MoS_2_, Figure S1a, Supporting Information). X‐ray diffraction (XRD) spectrum of the hybrid aerogel further confirms that the as‐deposited MoS_2_ possesses 2H phase (the peak of MoS_2_ (002) at 14.08°, Figure S1b, Supporting Information). At the same time, these spectra also indicate that the d‐spacing of Ti_3_C_2_T*_x_* aerogel washed by hydrochloric acid is less than that of Ti_3_C_2_T*_x_* aerogel because of Li^+^ ions among Ti_3_C_2_T*_x_* sheets being replaced by H^+^ ions. Furthermore, the d‐spacing of hybrid aerogel will be further decreased after ALD much slowly, due to the rest of water in the Ti_3_C_2_T*_x_* sheets being removed by heating.

The higher concentration of Ti_3_C_2_T*_x_* suspension can result in smaller lamellae spacing of the aerogels (Figure S2, Supporting Information) and more connecting “bridges” between the lamellae “walls” (yellow circles in Figure S2, Supporting Information), which can improve the conductive network and mechanical property of pristine Ti_3_C_2_T*_x_* aerogels. However, excessive Ti_3_C_2_T*_x_* concentration will generate conductive channel, thus resulting in the loss of MA property. According to experimental results, negative value will appear in the permittivity of the as‐produced aerogels (means no MA ability) when Ti_3_C_2_T*_x_* concentration is more than 40 mg mL^−1^ (FigureS3, Supporting Information). Comprehensively considering the requirements of mechanical properties, the pristine aerogel prepared with a Ti_3_C_2_T*_x_* concentration of 30 mg mL^−1^ has been selected as the templates for fabricating hybrid aerogels (Figure S4, Supporting Information), and the corresponding density and porosity are 36.8 mg cm^−3^ and 98.4%, respectively (Equation S1, Supporting Information).

**Figure 2:**
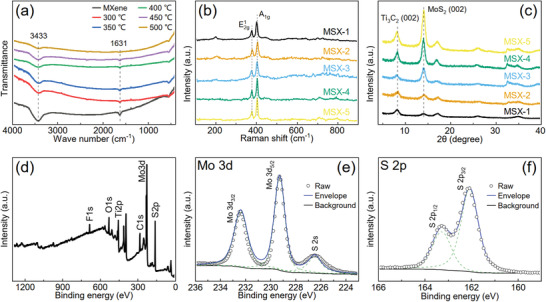
a) FTIR spectra of MX‐3 with different annealing temperatures. b) Raman spectra and c) XRD patterns of MoS_2_/MXene hybrid aerogels obtained from different ALD cycles. d) XPS survey, e) Mo 3d, and f) S 2p spectra of MSX‐1.

A large number of functional surface groups (such as OH) on Ti_3_C_2_T*_x_* aerogel which is identified by FTIR characteristic peaks (3433 cm^−1^, **Figure** **2**a) can facilitate the adsorption of ALD precursor molecules thus promoting the surface reactions. The deposition temperature in ALD can largely affect the number of active groups, which decreases under continuous heating but does not disappear completely even at 500 °C. Although low temperature helps to retain more surface groups, it is not conducive to the crystallization of MoS_2_ during ALD according to our previous work.^[^ ^49^, ^56^ ^]^ Comprehensively considering these advantages and disadvantages, 450 °C has been selected as the deposition temperature of MoS_2_. The intensities of the characteristic peaks in Raman (E^1^_2g_ and A_1g_, Figure 2b) and XRD (MoS_2_ (002), Figure 2c) for these samples increase as ALD cycle increases, which can confirm the increased MoS_2_ thickness. In addition, XRD data further indicate that ALD cycle only controls the thickness of MoS_2_ films instead of changing their polycrystalline structures.

The X‐ray photoelectron spectroscopy (XPS) survey of MSX‐1 reveals that the hybrid aerogels contain Ti, C, O, F, Mo, and S elements (Figure 2d). The characteristic peaks of Mo 3d doublet and S 2p doublet at the binging energy of 232.4 and 229.3 eV and 163.3 and 162.1 eV are observed in the corresponding high‐resolution spectra (Figure 2e,f), further confirming objective existence of MoS_2_. Moreover, the S/Mo ratio of the as‐deposited MoS_2_ on the Ti_3_C_2_T*_x_* aerogel is 1.75 instead of 2, indicating sulfur defects in the as‐deposited MoS_2_. These defects can generate abundant dipoles, which benefits to dissipating the energy of EM waves through polarization relaxation under the external alternating EM fields.

**Figure 3:**
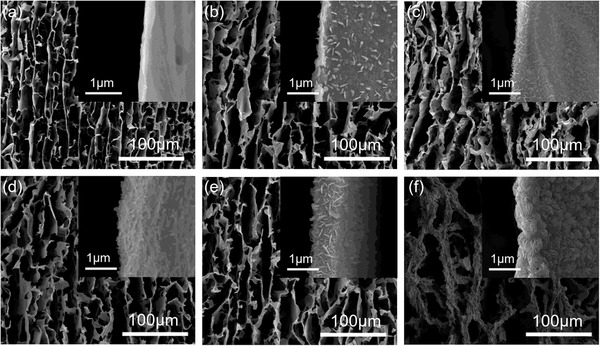
SEM images of the transverse section for a) the Ti_3_C_2_T*_x_* aerogel and b–f) the MoS_2_/MXene hybrid aerogels from 100 to 500 ALD cycles, respectively.

Scanning electron microscope (SEM) images of the aerogels (**Figure** **3**) obtained from 100 to 500 ALD cycles indicate that the conformal heterogeneous interfaces between MoS_2_ and Ti_3_C_2_T*_x_* have been successfully formed. As ALD cycle increases, discontinuous MoS_2_ nanoflakes gradually grow into continuous fiber texture and the layer thickness also increases. At the same time, the microporous structure of the aerogel templates is not damaged due to the excellent conformal feature of ALD. Whether viewed from the transverse direction (Figure 3) or the longitudinal direction (Figure S5a–f, Supporting Information), MoS_2_ has been uniformly deposited on the complex 3D surface of Ti_3_C_2_T*_x_* lamellae, and the as‐formed heterogeneous interfaces between MoS_2_ and Ti_3_C_2_T*_x_* completely inherit the original 3D structure of the templates.

Elements mapping images of MSX‐1 surface can confirm the uniformity of deposited MoS_2_ (Figure S5g, Supporting Information), where there is no obvious aggregation of Mo and S elements. In this way, the hydrophilic MXene aerogels are transformed into the hydrophobic MoS_2_/MXene hybrid aerogels by the ALD‐made MoS_2_ (Figure S6a, Supporting Information). Correspondingly, the contact angle of MoS_2_/MXene aerogels increases positively from 93.9° to 143.1° as ALD cycle increases from 100 to 500 (Figure S6b, Supporting Information), and the time to maintain contact angle also increases with ALD cycle increasing and finally tends to be constant at the ALD cycle of 400 or more (Figure S6c, Supporting Information). At the same time, the thermal conductivity of the hybrid aerogels is also improved (Figure S6d, Supporting Information). Specifically, sample MX‐3 (1.3 cm thickness) and MSX‐4 (1.3 cm thickness) need ≈10 and ≈8 s to reach thermal stability, respectively. The average real‐time temperature curves for MX‐3 and MSX‐4 aerogel at the bottom (Ar1), middle (Ar2), and top (Ar3) are ≈108, ≈64, and ≈51 °C and ≈115, ≈70, and ≈56 °C (Figure S6e,f, Supporting Information), respectively. These indicate that MSX‐4 can reach thermal stability faster and has a better thermal conductivity.

**Figure 4:**
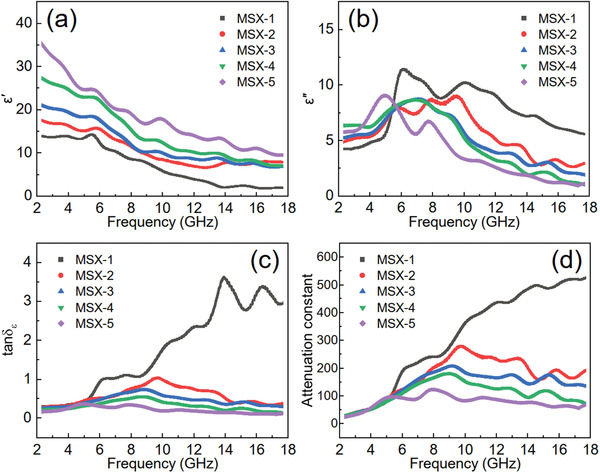
a) The real parts and b) imaginary parts of complex permittivity, c) dielectric loss tangent tan*δ_*ε*_*, and d) attenuation constant for MoS_2_/MXene hybrid aerogels with different ALD cycles, respectively.

EM absorbing materials can exhibit polarization and magnetization responses to external EM fields simultaneously, which are described by complex permittivity (*ε*_r_ = *ε*′ − *jε*″) and complex permeability (*μ*_r_ = *µ*′ − *jµ*″), respectively. Generally, the real parts (*ε*′ and *µ*′) represent the ability of storing EM energy, while the imaginary parts (*ε*″ and *µ*″) represent the ability of dissipating EM energy. Since there is no magnetic component in these samples, the value of *μ*_r_ is almost constant (1*–j*0, Figure S7, Supporting Information). In addition, *ε*′ and *ε*″ show obvious frequency‐dependent fluctuations, which is caused by dielectric relaxation according to Debye theory (Equations S4 and S5, Supporting Information). Correspondingly, the frequency dependence of the permittivity for MoS_2_/MXene hybrid aerogels obtained from different ALD cycles (**Figure** **4**a,b) indicate that the value of *ε*′ gradually decreases as the frequency increases from 2 to 18 GHz, while that of *ε*″ increases first then turns to decrease at the extreme point ≈6 GHz for all the samples.

According to general cognitions, *ε*′ and *ε*″ usually show similar changing trend, and *ε*″ is positively related to the conductivity of the materials.^[^ ^4^, ^57^ ^]^ However, our experimental results look different from those mentioned above. The average value of *ε*′ increases from 7.1 to 18.3 as ALD cycle increases from 100 to 500 in the frequency range of 2–18 GHz, due to the thickness increase of those ALD‐made MoS_2_.^[^ ^18^, ^58^, ^59^ ^]^ Meanwhile, the average value of *ε*″ shows an opposite changing trend which decreases from 7.6 to 4.2 as ALD cycle increases, which is caused by the changes of some internal factors that determine *ε*″. According to Equation S5, Supporting Information, *ε*″ depends mainly on the comprehensive contributions of the polarization loss (*ε*_p_″) and conductive loss (*ε*_c_″). The numerical change of *ε*_c_″ and *ε*_p_″ versus the detection frequency can be obtained by a nonlinear least squares fitting method,^[^ ^39^, ^60^ ^]^ which indicates that *ε*_c_″ is higher than *ε*_p_″ for the samples of MSX series (Figure S8a–e, Supporting Information). Furthermore, the average value of *ε*_p_″ increases slightly while that of *ε*_c_″ decreases dramatically as ALD cycle increases. Even so, *ε*_c_″ is also higher than *ε*_p_″ for those samples obtained under different ALD cycles (Figure S8f, Supporting Information). Therefore, the contribution of *ε*_c_″ to *ε*″ is much higher than that of *ε*_p_″ and the electrical conductivity is a dominant factor for *ε*″, which can be further demonstrated by the consistency between *ε*″ and the changing trend of DC conductivity (Figure S9, Supporting Information) as ALD cycle increases. In addition, the samples with disrupted network (for example, samples containing smashed MSX‐3 with filling ratio of 96.1 wt%, Figure S10, Supporting Information) fail to form sufficient and continuous electronic conduction path, which causes the value of *ε*″ for the disordered sample to be significantly reduced (Figure S11, Supporting Information). Therefore, when MoS_2_ is deposited on the surface of 3D aerogel, the conformal heterogeneous interfaces can cause the conductivity of hybrid to decrease with ALD cycle increasing, resulting in a seemingly abnormal decreasing trend of *ε*″.

The dielectric loss tangent (tan*δ_*ε*_*, Equation S2, Supporting Information, and Figure 4c) possesses similar trends with *ε*″ except for MSX‐1, which may be caused by the poor interface impedance matching resulted from the discontinuous MoS_2_ fakes on the surface of Ti_3_C_2_T*_x_* lamellae. Besides, the fluctuation trends of complex permittivity indicate the multiple polarization relaxation, which can be represented by the Cole–Cole semicircle according to Debye relaxation (Equation S6, Supporting Information). Several semicircles in the curve of *ε*′ versus *ε*″ (Figure S12, Supporting Information) indicate the multiple polarization relaxation, which can enhance the dissipation ability of EM waves. The attenuation constant *α* (Equation S7, Supporting Information) used for evaluating the loss capability of EM absorbing materials possesses a similar changing trend to tan*δ_*ε*_* (Equation S8, Supporting Information). The corresponding experimental results show that the attenuation constant decreases as ALD cycle increases (Figure 4d), indicating that Ti_3_C_2_T*_x_* lamellae can greatly attenuate the EM waves and increase the loss capability.

**Figure 5:**
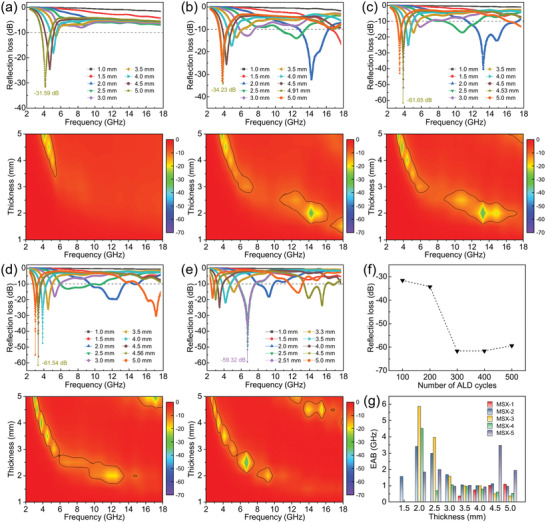
EM wave reflection loss and the corresponding 2D contour plots for a) MSX‐1, b) MSX‐2, c) MSX‐3, d) MSX‐4, and e) MSX‐5, respectively. f) RL_min_ values of MoS_2_/MXene aerogel at different number of ALD cycles. g) Effective absorption bandwidth histogram of MoS_2_/MXene hybrid aerogels.

The EM absorption performance of these hybrid aerogels can be described by the reflection loss (RL) values (Equation S9, Supporting Information). The frequency region where the RL value is less than −10 dB is usually termed the effective absorption bandwidth (EAB), corresponding to 90% of the incident EM being absorbed. The calculated reflection loss curves, corresponding 2D contours, and 3D plots of MoS_2_/MXene hybrid aerogels (**Figure** **5**a–e and Figure S13, Supporting Information) indicate that MSX‐3 displays the minimum *RL* value of −61.65 dB at the thickness of 4.53 mm and the maximum EAB value of 5.9 GHz at the thickness of 2.0 mm. Comparatively, the *RL* values of MX‐3 without MoS_2_ are always above −10 dB from 2 to 18 GHz (Figure S14a, Supporting Information). Although the absorption performance of MX‐3 by annealing at 450 °C can be improved slightly, the maximum *RL* value is still only −18.62 dB at the thickness of 1.5 mm (Figure S14b, Supporting Information), which indicates that pristine Ti_3_C_2_T*_x_* aerogels possess poor MA properties. Besides, the absorption performance of samples containing smashed MSX‐3 (96.1, 90, 80, and 70 wt%, Figure S11, Supporting Information) slightly increases with the filling ratio decreasing (MSX‐3 load ratio increasing). The porous structure of the aerogel is damaged in large quantities so that the absorption performance of the material will be greatly weakened. Compared with the related results reported by other groups, the absorbing properties of MoS_2_/MXene hybrid aerogels also seems relatively better (Table S2, Supporting Information). Moreover, RL_min_ value of these samples decreases first and then increases with ALD cycle increasing and then reaches a minimum value at 300 ALD cycles (Figure 5f), indicating that the modulation of the absorbing properties of the hybrid aerogels can be realized by the precise control in ALD fabrication. In addition, when the thickness is more than 2 mm, almost all of these hybrid aerogels show EAB (Figure 5g). Similarly, the EAB values decrease as the thickness increases and tend to be constant for all samples.

According to the previous analysis, although the attenuation constant decreases with ALD cycle increasing, there is no similar trend for EM absorption. RL_min_ value decreases first and then increased because the MA performance of these samples will also be affected by the impedance matching. Here, a delta‐function method is employed to express the impedance matching degree (Equation S12, Supporting Information), by which the delta value maps of MoS_2_/MXene hybrid aerogels with different ALD cycles are calculated and displayed (Figure S15, Supporting Information). Obviously, the area corresponding to a lower delta value increases with ALD cycle increasing, indicating that the heterogeneous interfaces can optimize the impedance matching effectively and achieve the optimal absorption performance at 300 ALD cycles.

**Figure 6:**
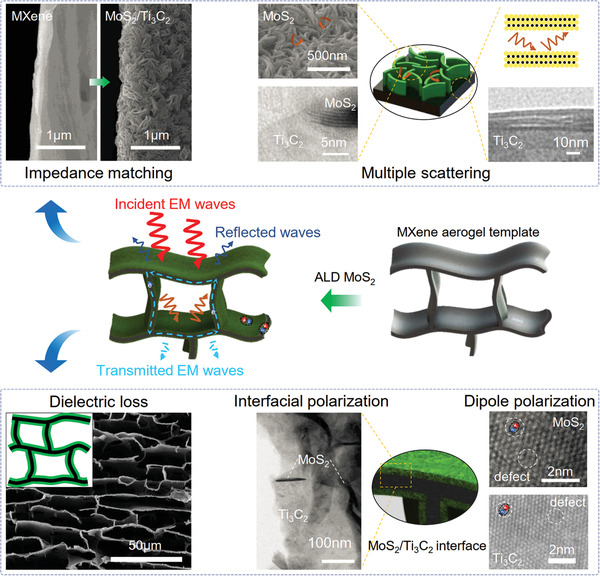
Schematic illustration of microwave absorption mechanisms for the MoS_2_/MXene aerogel.

The conformal heterogeneous interfaces on pristine Ti_3_C_2_T*_x_* aerogel template can transform its response to EM waves from shielding into absorbing. The impedance matching can be optimized by MoS_2_ conformally deposited on aerogels, ensuring more EM waves entered the absorber instead of being reflected. The integration of porous structure of these aerogels and excellent absorbing materials (MXene and MoS_2_) can enhance absorbing performance, ensuring that the incident EM waves will be tremendously absorbed (**Figure** **6**). On the other hand, the EMI shielding performances of pristine Ti_3_C_2_T*_x_* aerogels also increased as the concentration of MXene increased due to the EMI shielding specialty of itself (Figure S16a, Supporting Information). For MXene aerogels, the absorption efficiency (SE_A_) is higher than the reflection efficiency (SE_R_) (Figure S16b, Supporting Information), and further reduction of reflection will help to improve the absorption performance, which can be achieved by building special smart nanostructures. The ALD‐made heterogeneous interfaces between MoS_2_ and Ti_3_C_2_T*_x_* can optimize the impedance matching condition between MoS_2_ and the free space, which is confirmed by their delta value maps showed in Figure S15, Supporting Information. As ALD cycle increases, the area of lower delta value increases, indicating that the impedance matching has been effectively optimized by the heterogeneous interface, which will reduce the reflection of EM waves and allow more incident EM waves to pass through.

After the incident EM waves enter, they can be extended by multiple reflections in the distinctively hierarchical microporous structure of the hybrid aerogels. Meanwhile, multiple scattering will occur among the multilayer fiber texture MoS_2_ and Ti_3_C_2_T*_x_* when the incident EM waves penetrate its lamellae walls. Undoubtedly, the multiple reflection and scattering can prolong the propagation path of EM waves and are conducive to the dissipation of EM energy. The conductivity for the porous networks is gradually decreased with ALD‐made MoS_2_ increasing (Figure S9, Supporting Information), which is not advantageous to the conduction loss in the EM wave attenuation. However, the dielectric loss induced by interfacial polarization and dipole polarization has been significantly strengthened due to the ALD‐made MoS_2_ and its conformal heterointerfaces. More specifically, a capacitor‐like structure can be produced at the heterogeneous interfaces,^[^ ^61^, ^62^ ^]^ which contribute to the enhanced EM absorption due to the accumulation and oscillation of the charges under alternating EM fields. In addition, there exist many defects in MoS_2_ and Ti_3_C_2_T*_x_*, which can provide dipole polarization followed by polarization relaxation in an alternating EM field and further contribute to the enhanced EM waves dissipation.

## 3 Conclusion

In summary, pristine Ti_3_C_2_T*_x_* MXene aerogel templates were prepared by a multistep procedure combined with ice‐template, thawing process, and vacuum freeze‐drying, based on which MoS_2_/MXene hybrid aerogels were obtained via conformally depositing MoS_2_ by controlled ALD. The MA performance of the obtained aerogels was remarkably improved by the smart combinations of special microstructures and materials. The porous templates preserved its microstructure during the ALD and extended the reflection and scattering path of EM waves, while the ALD‐made MoS_2_ and its conformal heterointerface optimized the impedance matching. In addition, their preferable mechanical and hydrophobicity property was conducive to further practical applications.

## 4 Experimental Section

### Materials

Ti_3_AlC_2_ (MAX phase) powders (400 mesh, 11 Technology Co., Ltd.), LiF (AR, Aladdin), HCl (36–37% w/w, Sinopharm Chemical Reagent Co., Ltd.), MoCl_5_ (99.6%, Aladdin), H_2_S (99.99%, Nanjing Shangyuan Industrial Gas Factory), N_2_ (99.99%, Nanjing Special Gas Factory Co., Ltd.) were used.

### Synthesis of Ti_3_C_2_T*_x_* MXene Dispersion

Ti_3_AlC_2_ powders were etched with the method reported previously.^[^ ^63^, ^64^ ^]^ LiF (9.6 g) was added to HCl (120 mL, 9 m) under magnetic stirring for 5 min in a 500 mL of PTFE beaker. Ti_3_AlC_2_ powders (6 g) were gradually added to the etchant, and the mixture was further stirred for 36 h at room temperature. The acidic mixture was washed with deionized H_2_O via centrifugation (10 min per cycle at 2000 × *g* centrifugal force) for multiple cycles until the supernatant became neutral (pH > 5). Mild sonication was implemented for 20 min and the bath temperature was controlled below 25 °C. Ti_3_C_2_T*_x_* dispersion was centrifuged at 1000 × *g* for 20 min to remove the impurities. After that, Ti_3_C_2_T*_x_* slurry was collected by centrifugation (45 min, 12 000 × *g*), which was diluted to 50, 40 30, 20, and 10 mg mL^−1^ of Ti_3_C_2_T*_x_* dispersion, respectively.

### Preparation of Ti_3_C_2_T*_x_* Aerogel Templates

The mold filled with Ti_3_C_2_T*_x_* dispersion was placed on the top of copper block in liquid nitrogen until Ti_3_C_2_T*_x_* dispersion was completely frozen. Ti_3_C_2_T*_x_* ice gel was separated from the mold and stored in the refrigerator (−20 °C) for 4 h until the temperature of ice gel was stabilized. Ti_3_C_2_T*_x_* ice gel was directly placed in HCl solution (5 m) for 8 h to complete thawing. Then HCl solution was replaced by deionized water for several times until pH > 5. The pristine Ti_3_C_2_T*_x_* aerogel was obtained by rapidly freezing Ti_3_C_2_T*_x_* hydrogel in liquid nitrogen and freeze‐dried at −70 °C for 48 h.

### Fabrication of MoS_2_/MXene hybrid Aerogels

The pristine Ti_3_C_2_T*_x_* aerogels were treated by plasma with 120 s (PCE‐6). These treated samples were placed in a commercial ALD setup (SUNALETMR‐100, PICOSUN). MoCl_5_ and H_2_S precursors were alternately injected into the ALD chamber under N_2_ flow as carrier gas (flow rate of 50 sccm). The dosing conditions for MoCl_5_ were 2 s of exposure time and 30 s of N_2_ purging time; for H_2_S: 1 s of exposure time and 30 s of N_2_ purging time. The temperature in the ALD chamber was kept at 450 °C.

### Characterizations

Raman spectroscopy (Xper‐Ram C, Nanobase) was used in a 532 nm laser. XRD (Smartlab‐3, Rigaku) was carried out using Cu K*α* radiation (*λ* = 1.54 Å) at 35 mA and 50 kV. SEM (Inspect F50, FEI) and transmission electron microscope (G220, FEI) were applied for microscopic observations. XPS (PreVac) was utilized for analyzing the bonding characteristics and chemical composition of the samples. The contact angle meter (JC2000C1, POWEREACH) was employed for hydrophobicity test with a deionized water droplet (1 µL). The thermal analysis was implemented using a thermal imaging camera (FOTRIC 343). The electrical conductivity was measured by a four‐point probe device (HPS2662).

### Electromagnetic Parameter Test

A vector network analyzer (Ceyear 3656D) was employed to measure the EM parameters of the aerogels in the frequency range of 2–18 GHz on the basis of a coaxial flange method. The molten paraffin was slowly impregnated into the mold until the aerogel samples were completely immersed (Figure S17, Supporting Information). The composites were kept in a vacuum oven at 80 °C for 10 min to ensure that the molten paraffin could remove air bubbles and better fill the pores in the aerogel. After cooling sufficiently at room temperature, the cured composites were cut into coaxial ring shape with an inner diameter of 3.04 mm and an outer diameter of 7.0 mm for the EM parameter test. The mass fraction of paraffin in the composites was calculated by comparing the mass of samples before and after aerogel filling, and it should be noted that the over‐flowing paraffin outside the aerogel boundary was carefully removed for accurate quantification. Thus the filling ratio of paraffin *R* = 1 − *M*_1_ (the mass of aerogel)/*M*_2_ (the mass of composites). Therefore, the filling ratios of MSX samples obtained from 100 to 500 ALD cycles were 96.8, 96.4, 96.1, 95.8, and 95.4 wt%, respectively (Table S3, Supporting Information). In addition, the samples with certain filling ratio were prepared by quantitatively mixing the smashed aerogel powders and paraffin.

### Statistical Analysis

The bar plots were presented as mean ± SD, and the data were measured at least three repeats. The high‐resolution XPS spectra were fitted using the program XPSPEAK (Version 4.1). The data were analyzed and plotted using the software Origin (OriginLab Corp.).

## Conflict of Interest

The authors declare no conflict of interest.

## Supporting information

Supporting Information

## Data Availability

The data that support the findings of this study are available from the corresponding author upon reasonable request.
