# Supplementary material for: MoS2/MXene Aerogel with Conformal Heterogeneous Interfaces Tailored by Atomic Layer Deposition for Tunable Microwave Absorption
Source: Adv Sci (Weinh). 2022 Jan 23;9(7):2101988. doi: 10.1002/advs.202101988 (PMC8895119; doi:10.1002/advs.202101988)
Supplement: Supplementary file 1 — Supporting Information [file ADVS-9-2101988-s001.pdf]

ADVANCED SCIENCE
Open Access

Supporting Information

for *Adv. Sci.,* DOI: 10.1002/advs.202101988

MoS_2/MXene Aerogel with Conformal Heterogeneous Interfaces Tailored by Atomic Layer Deposition (ALD) for Tunable Microwave Absorption

*Junjie Yang, Jianqiao Wang, Huiqin Li, Ze Wu, Youqiang Xing, Yunfei Chen and Lei Liu**

Supporting Information

MoS_2/MXene Aerogel with Conformal Heterogeneous Interfaces Tailored by Atomic Layer Deposition (ALD) for Tunable Microwave Absorption

*Junjie Yang, Jianqiao Wang, Huiqin Li, Ze Wu, Youqiang Xing, Yunfei Chen and Lei Liu**

J. Yang, J. Wang, H. Li, Z. Wu, Y. Xing, Prof. Y. Chen, Prof. L. Liu,
School of Mechanical Engineering, Southeast University, Nanjing 211189, People's Republic of China
E-mail: liulei@seu.edu.cn

Keywords: atomic layer deposition, MoS_2 film, aerogel, heterogeneous interface, microwave absorption

(1) Porosity of aerogel is calculated by the equation as following:[1, 2]

$$\eta = 1 - \frac{\left(\frac{m_1}{\rho_1} + \frac{m_2}{\rho_2}\right)}{V_0} \qquad \text{(S1)}$$

where η is the porosity of aerogels, m_1 and m_2 are the mass of $Ti_3C_2T_x$ and MoS_2, ρ_1 and ρ_2 are the density of $Ti_3C_2T_x$ (2.39 g cm^{-3}) and MoS_2 (4.8 g cm^{-3}), V_0 is the volume of the sample.

(2) Tangent of loss angle δ_ε and δ_μ is expressed by the equation as following:[3, 4]

$$tan\delta_\varepsilon = \varepsilon''/\varepsilon', tan\delta_\mu = \mu''/\mu' \qquad \text{(S2)}$$

where δ_ε and δ_μ are the dielectric loss angle and magnetic loss angle, ε' and ε'' are the real parts and the imaginary parts of complex permittivity ($\varepsilon_r = \varepsilon' - j\varepsilon''$), μ' and μ'' are the real parts and the imaginary parts of complex permeability ($\mu_r = \mu' - j\mu''$).

(3) Cole-Cole circle

The relative complex permittivity can be expressed by the following equation, according to Debye theory: [5, 6]

$$\varepsilon_r = \varepsilon' - j\varepsilon'' = \varepsilon_\infty + \frac{\varepsilon_s - \varepsilon_\infty}{1 + j2\pi f\tau} \qquad \text{(S3)}$$

where ε_s is the static dielectric constant, ε_∞ is the dielectric constant at infinite frequency, and τ is the polarization relaxation time, respectively. Then the ε' and ε'' can be deduced into:

$$\varepsilon' = \varepsilon_\infty + \frac{\varepsilon_s - \varepsilon_\infty}{1+(2\pi f\tau)^2} \tag{S4}$$

$$\varepsilon'' = \frac{2\pi f\tau(\varepsilon_s - \varepsilon_\infty)}{1+(2\pi f\tau)^2} + \frac{\sigma}{2\pi f\varepsilon_0} = \varepsilon_p'' + \varepsilon_c'' \tag{S5}$$

where ε_p'' is the polarization loss, and ε_c'' is the conductive loss. The relationship between ε' and ε'' can be written as:

$$\left(\varepsilon' - \frac{\varepsilon_s+\varepsilon_\infty}{2}\right)^2 + (\varepsilon'')^2 = \left(\frac{\varepsilon_s-\varepsilon_\infty}{2}\right)^2 \tag{S6}$$

In the curve of $\varepsilon' - \varepsilon''$, every single semicircle was called as the Cole-Cole semicircle,[5] corresponding to the Debye dipolar relaxation.

(4) Attenuation constant is expressed by the equation as following:[7, 8]

$$\alpha = \frac{\sqrt{2}\pi f}{c} \times \sqrt{(\mu''\varepsilon'' - \mu'\varepsilon') + \sqrt{(\mu''\varepsilon'' - \mu'\varepsilon')^2 + (\mu'\varepsilon'' + \mu''\varepsilon')^2}} \tag{S7}$$

This equation can be expressed by tangent of loss angle, as following:

$$\alpha = \frac{\sqrt{2}\pi f}{c}\sqrt{\mu'\varepsilon'}\sqrt{(tan\delta_\varepsilon tan\delta_\mu - 1) + \sqrt{(tan\delta_\varepsilon tan\delta_\mu - 1)^2 + (tan\delta_\varepsilon + tan\delta_\mu)^2}} \tag{S8}$$

where α is the attenuation constant, c is the speed of light, and f is the frequency of electromagnetic wave.

(5) The reflection loss is calculated according to the transmission line theory,[7, 9, 10] as following:

$$RL = 20\, log\left|\frac{Z_{in}-Z_0}{Z_{in}+Z_0}\right| \tag{S9}$$

$$Z_{in} = Z_0\sqrt{\frac{\mu_r}{\varepsilon_r}}\,tanh\left[j\frac{2\pi fd}{c}\sqrt{\mu_r\varepsilon_r}\right] \tag{S10}$$

where RL is the reflection loss, Z_{in} is the input characteristic impedance, Z_0 is the impedance of free space, and d is the thickness of the aerogel.

The normalized input impedance (Z) is expressed by the equation, as following:

$$Z = \left|\frac{Z_{in}}{Z_0}\right| = \left|\sqrt{\frac{\mu_r}{\varepsilon_r}} tanh\left[j\frac{2\pi fd}{c}\sqrt{\mu_r\varepsilon_r}\right]\right| \quad \text{(S11)}$$

(6) The impedance matching is calculated by a delta-function method,[5] as following:

$$\Delta = |sinh^2(Kfd) - M| \quad \text{(S12)}$$

where K and M are determined by the ε_r and μ_r:

$$K = \frac{4\pi\sqrt{\mu'\varepsilon'}\, sin\frac{\delta_e+\delta_m}{2}}{c\, cos\,\delta_e\, cos\,\delta_m} \quad \text{(S13)}$$

$$M = \frac{4\mu'\, cos\,\delta_e \varepsilon'\, cos\,\delta_m}{(\mu' cos\,\delta_e - \varepsilon'\, cos\,\delta_m)^2 + \left[tan\left(\frac{\delta_m}{2} - \frac{\delta_e}{2}\right)\right]^2 (\mu'\, cos\,\delta_e + \varepsilon'\, cos\,\delta_m)^2} \quad \text{(S14)}$$

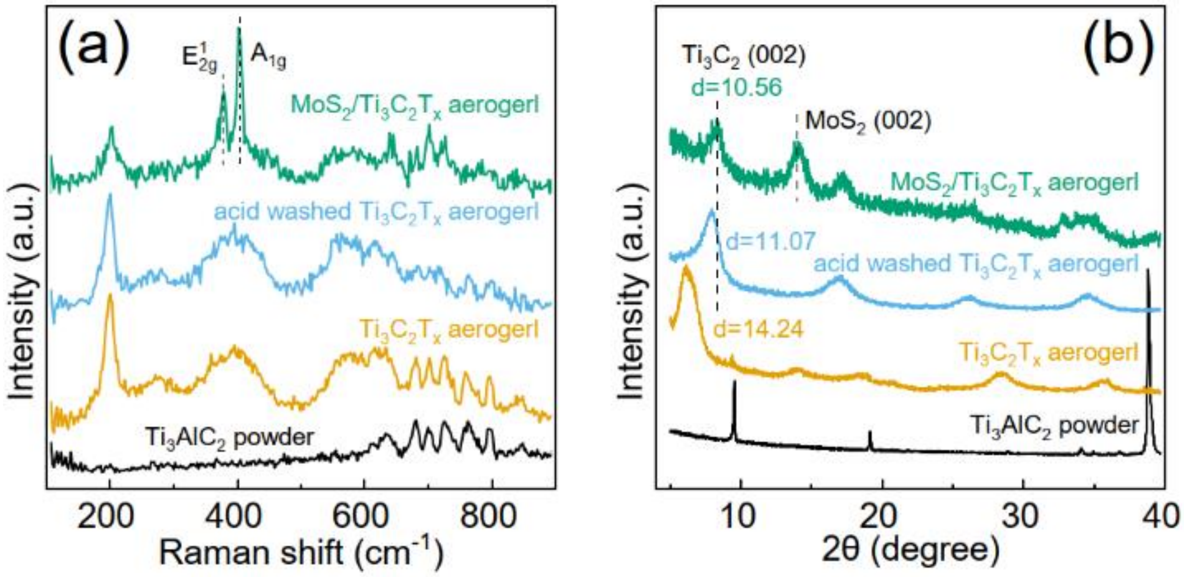

Figure S1. (a) Raman spectra and (b) XRD patterns of the samples during preparation of hybrid aerogel.

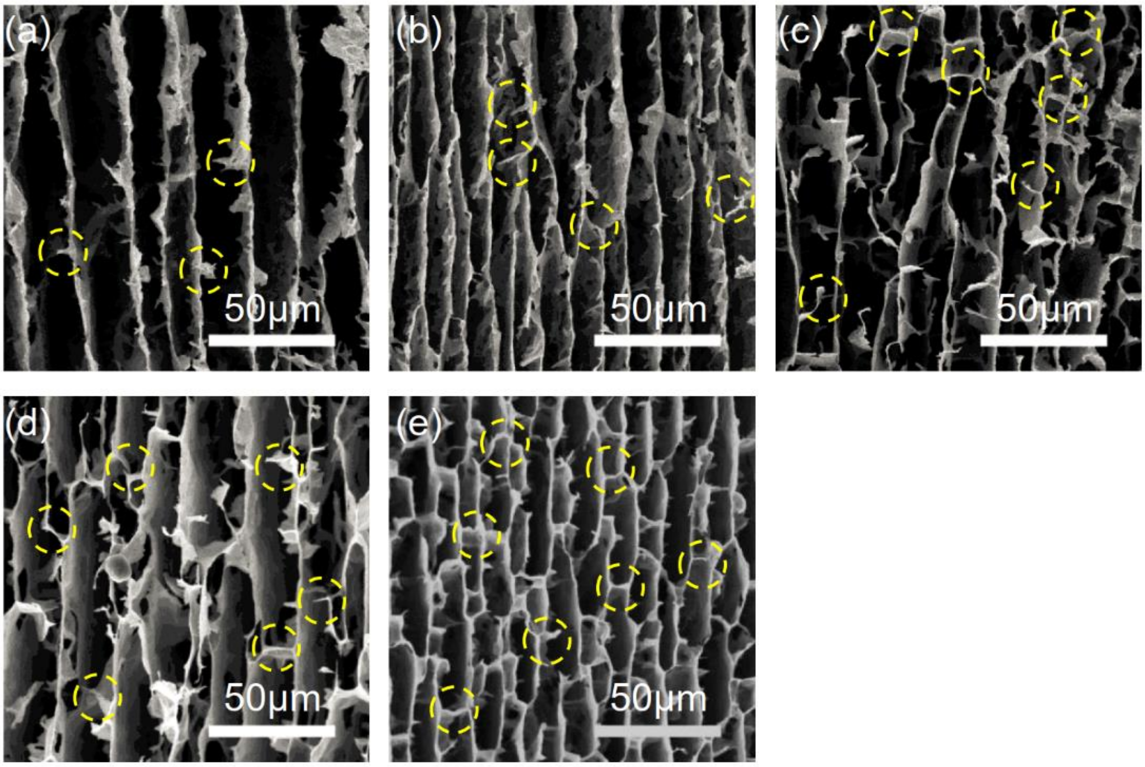

Figure S2. SEM images of $Ti_3C_2T_x$ aerogels prepared from suspension with different concentrations (a) 10 mg mL^{-1}, (b) 20 mg mL^{-1}, (c) 30 mg mL^{-1}, (d) 40 mg mL^{-1}, and (e) 50 mg mL^{-1}.

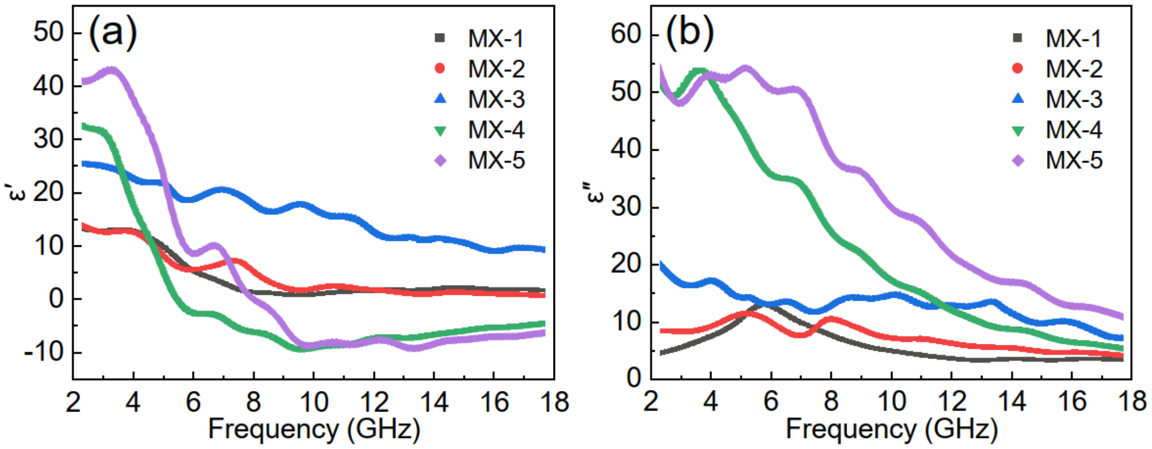

Figure S3. (a) Real parts and (b) imaginary parts of complex permittivity for $Ti_3C_2T_x$ aerogels with different concentrations suspensions.

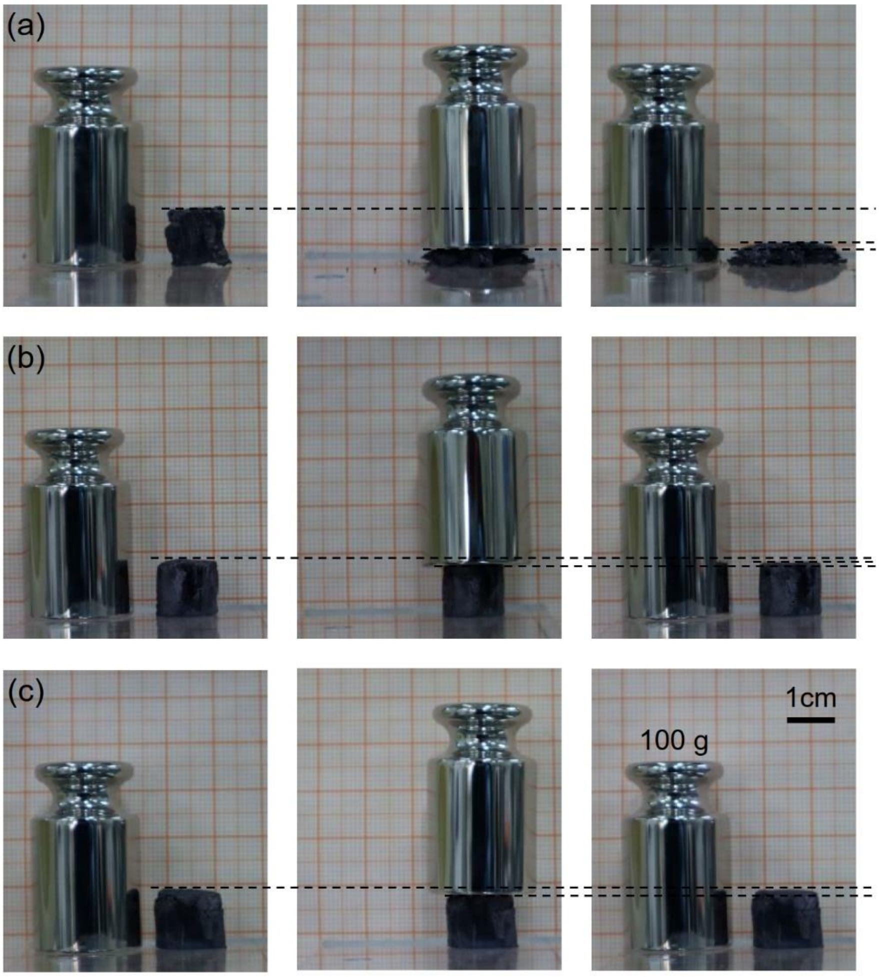

Figure S4. Digital images of the compression test of the sample (a) MX-1, (b) MX-2, and (c) MX-3.

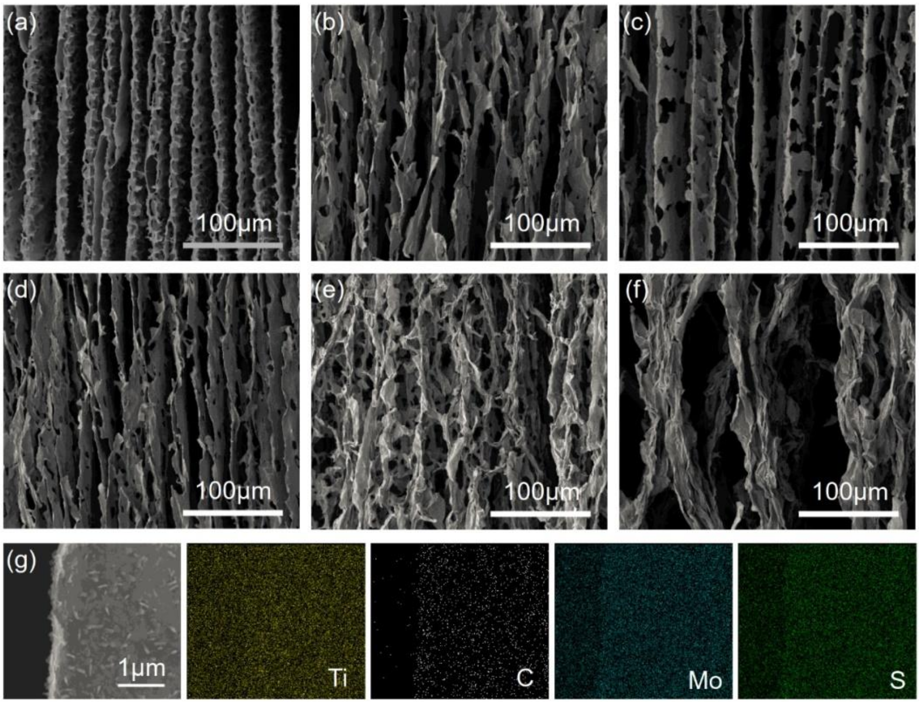

Figure S5. SEM images of the longitudinal section for (a) MX-3 (b) MSX-1, (c) MSX-2, (d) MSX-3, (e) MSX-4, and (f) MSX-5. (g) Elements mapping images of MSX-1.

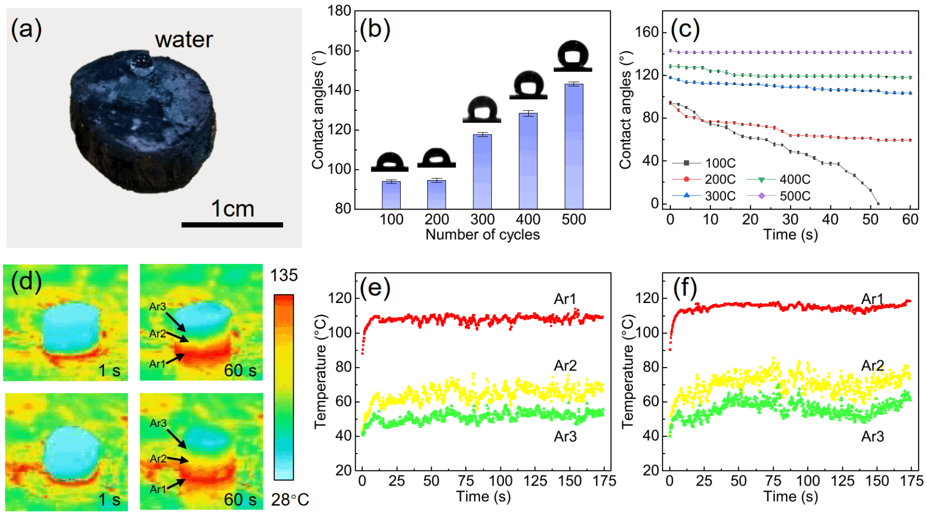

Figure S6. (a) Digital image of water droplet on an MSX-4 aerogel. (b) The initial contact angle of MoS_2/MXene aerogels with different ALD cycles. (c) The variation of contact angle of MoS_2/MXene aerogels with increasing time. (d) Infrared thermal images of MXene aerogel (MX-3, up) and MoS_2/MXene aerogel (MSX-4, down) on a heating platform. The temperature-time curves of the three different areas for (e) MXene aerogel (MX-3) and (f) MoS_2/MXene aerogel (MSX-4).

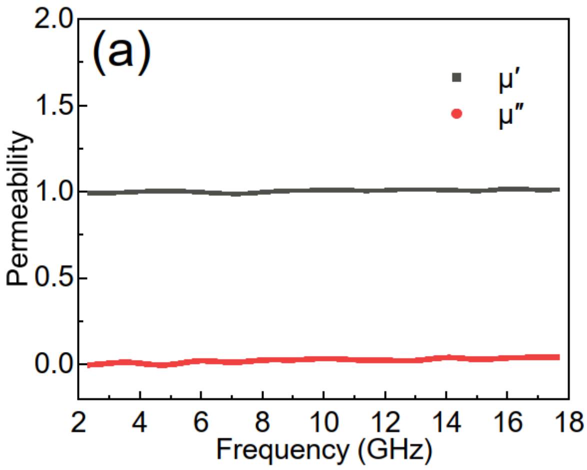

Figure S7. Complex permeability of (a) $Ti_3C_2T_x$ aerogel and (b) MoS_2/MXene hybrid aerogel, respectively.

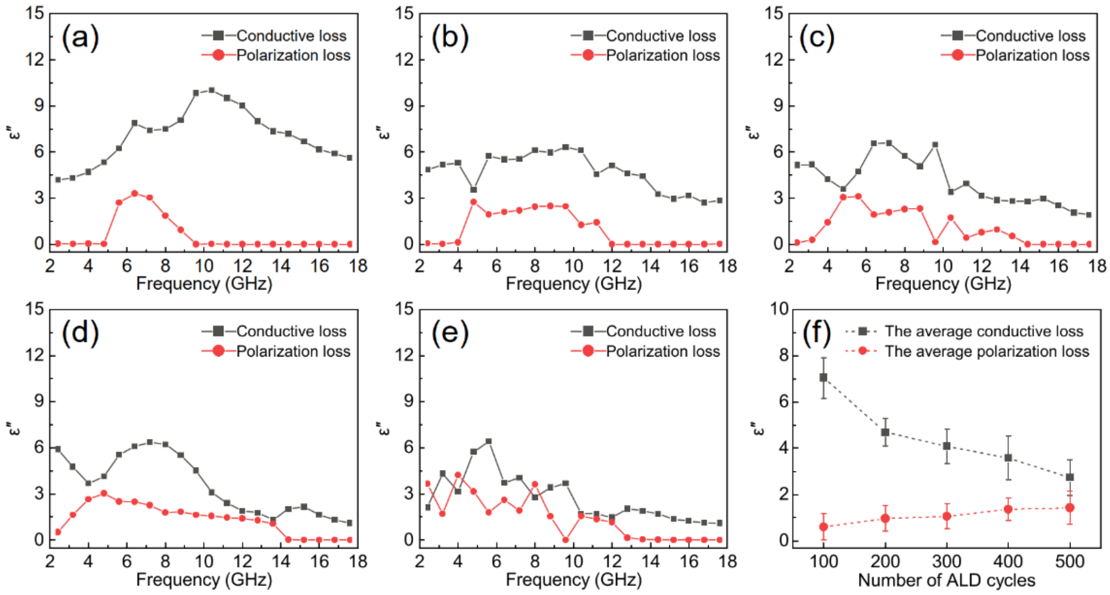

Figure S8. The fitted ε_c" and ε_p" versus frequency of (a) MSX-1, (b) MSX-2, (c) MSX-3, (d) MSX-4, and (e) MSX-5, respectively. (f) The fitted averages of ε_c" and ε_p" of the samples from 100 to 500 ALD cycles. The ε_c" and the ε_p" curves were fitted and obtained by non-linear least squares fitting method provided in the reference [11,12].

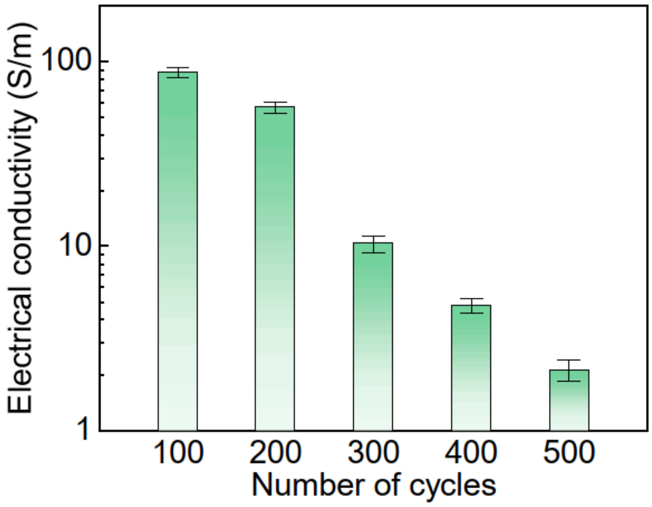

Figure S9. The electrical conductivity of MoS_2/MXene aerogels with the number of ALD cycles from 100 to 500 cycles.

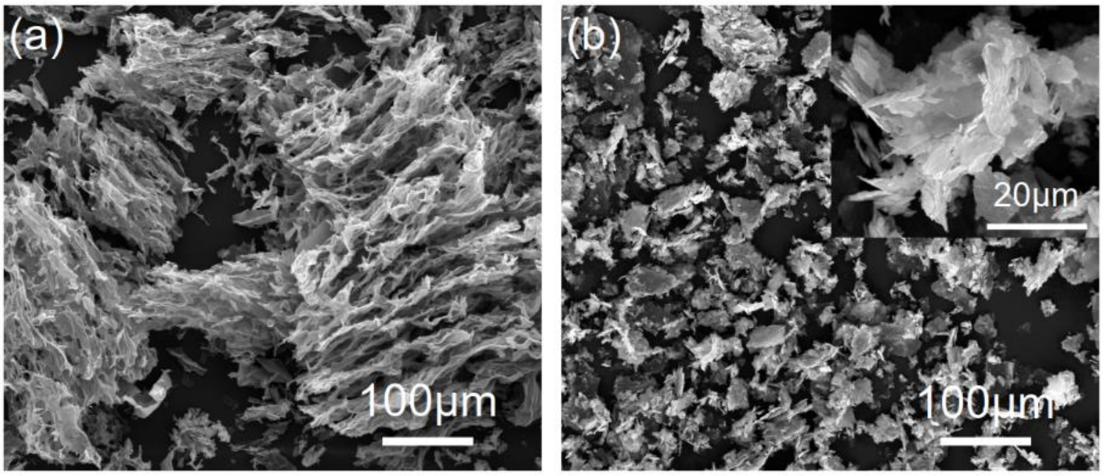

Figure S10. The SEM images of the smashed MSX-3 aerogel samples. The aerogel samples were first broken using a knife, and then ground in the mortar for 1 minute. (a) was the SEM image of the broken sample. (b) was the SEM image of the ground sample.

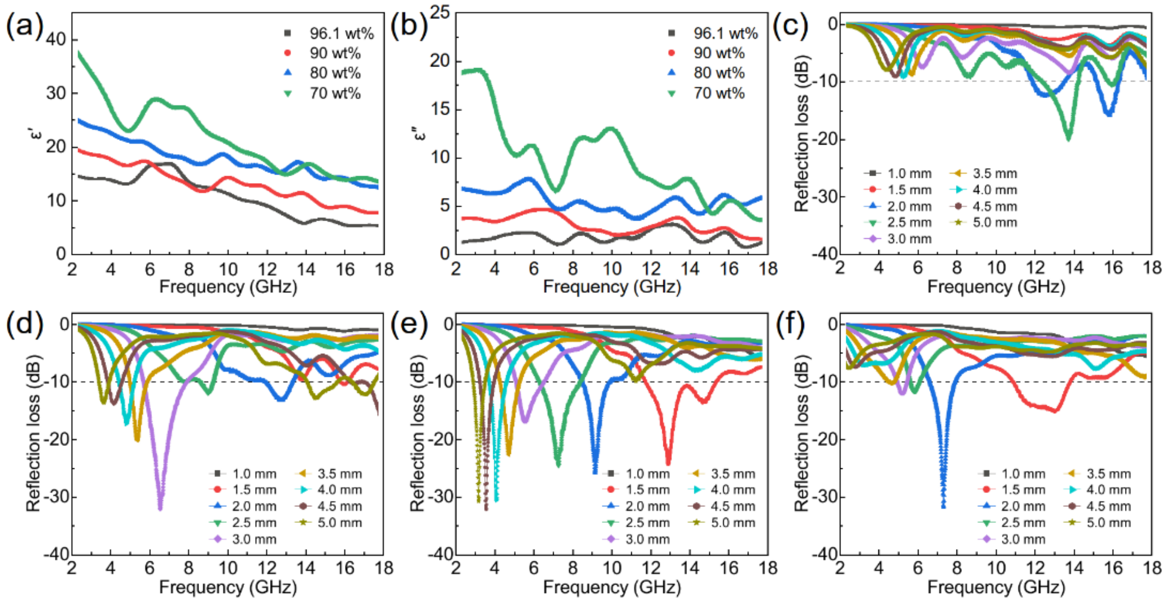

Figure S11. (a) Real parts and (b) imaginary parts of complex permittivity for the smashed MSX-3 samples with different filling ratio. The reflection loss curves of smashed MSX-3 samples with filling ratio of (c) 96.1 wt%, (d) 90 wt %, (e) 80 wt %, (f) 70 wt %, respectively.

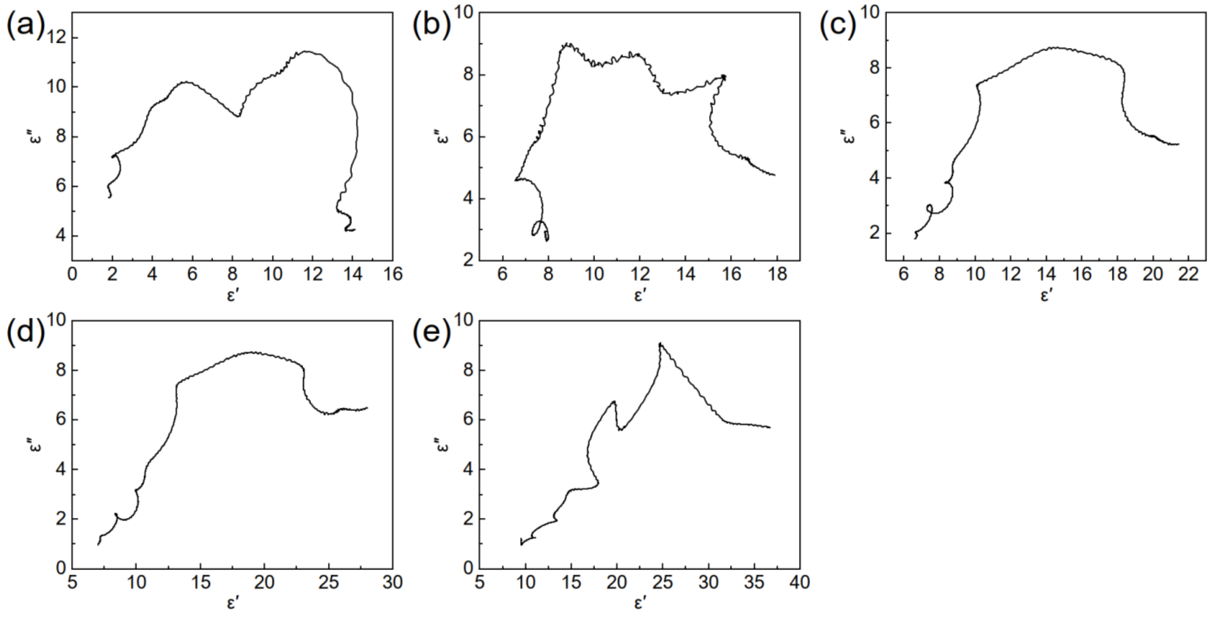

Figure S12. Cole-Cole curves of (a) MSX-1, (b) MSX-2, (c) MSX-3, (d) MSX-4, and (e) MSX-5, respectively.

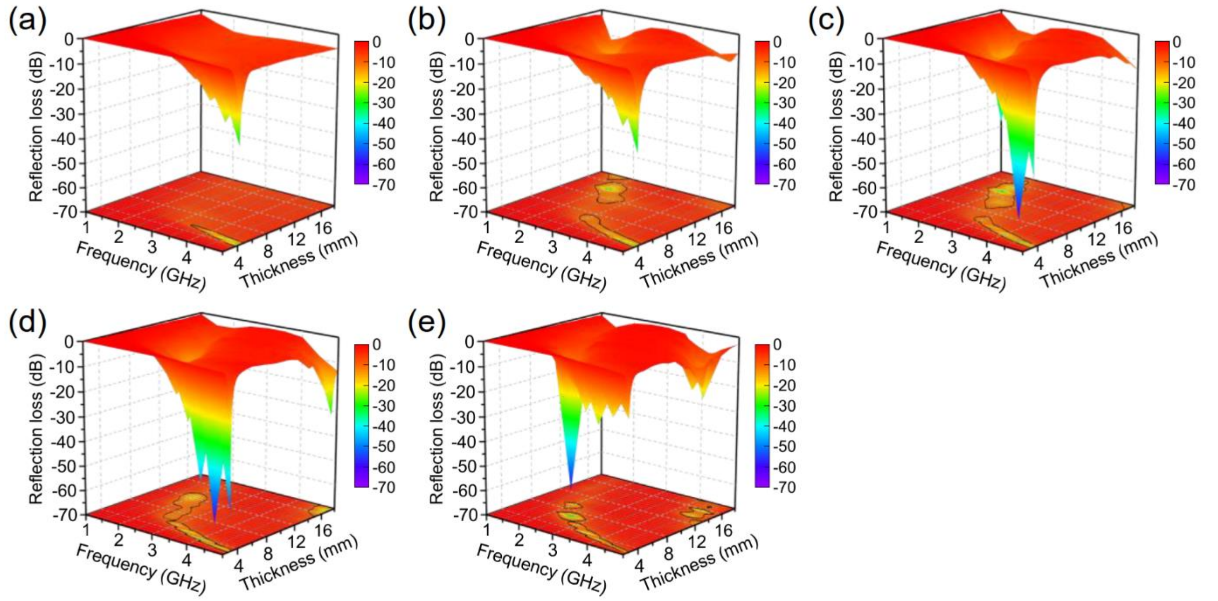

Figure S13. 3D RL curves of (a) MSX-1, (b) MSX-2, (c) MSX-3, (d) MSX-4, and (e) MSX-5, respectively.

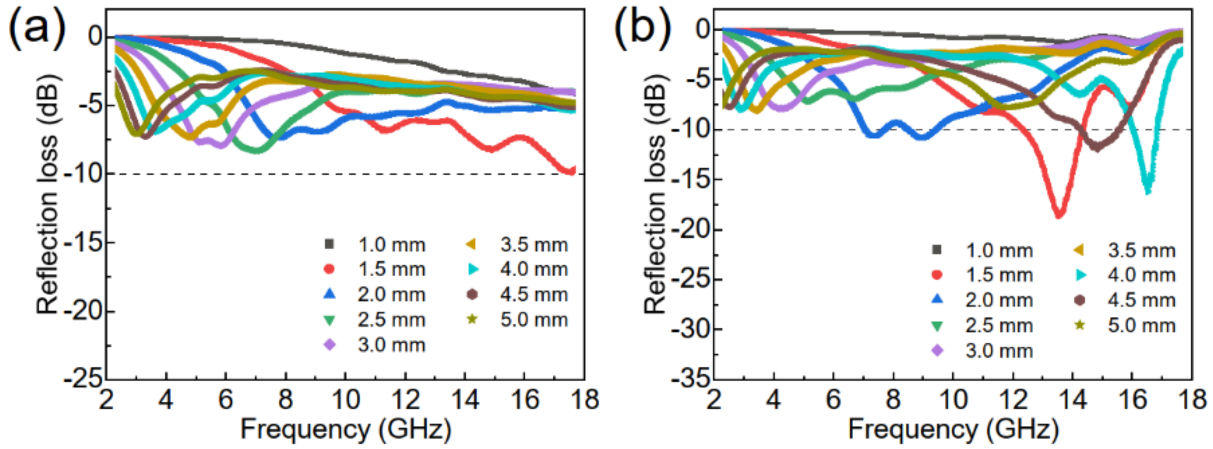

Figure S14. The reflection loss curves of (a) MX-3 and (b) MX-3 with 450 °C of annealing temperatures.

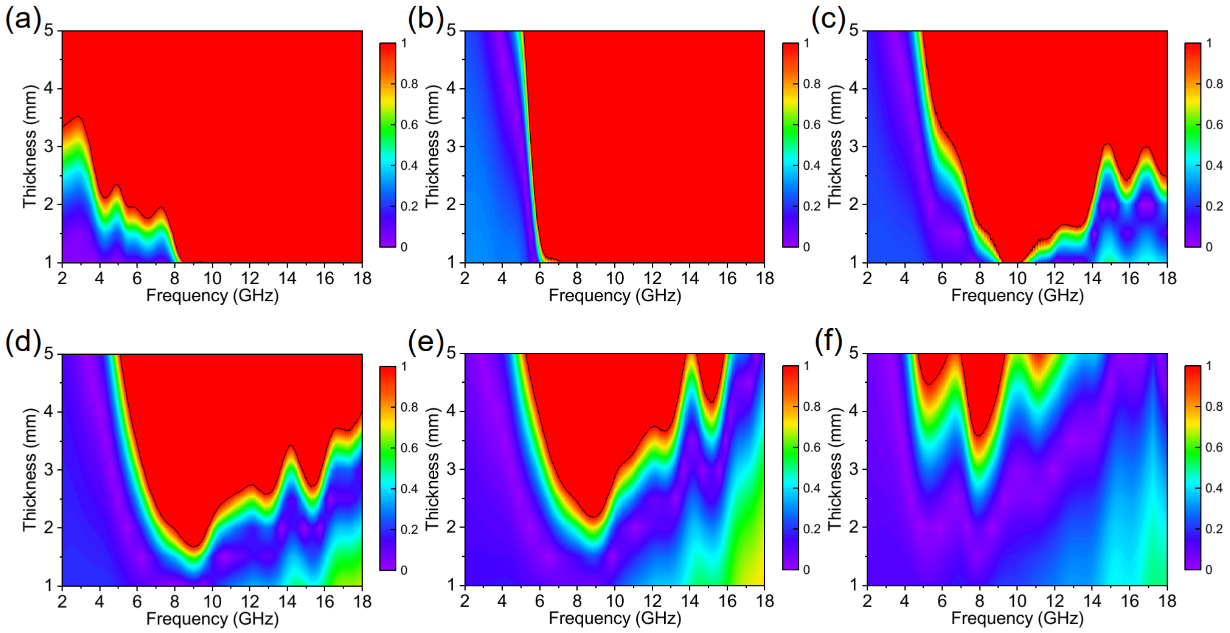

Figure S15. Delta value mappings of (a) MX-3, (b) MSX-1, (c) MSX-2, (d) MSX-3, (e) MSX-4, and (f) MSX-5, respectively.

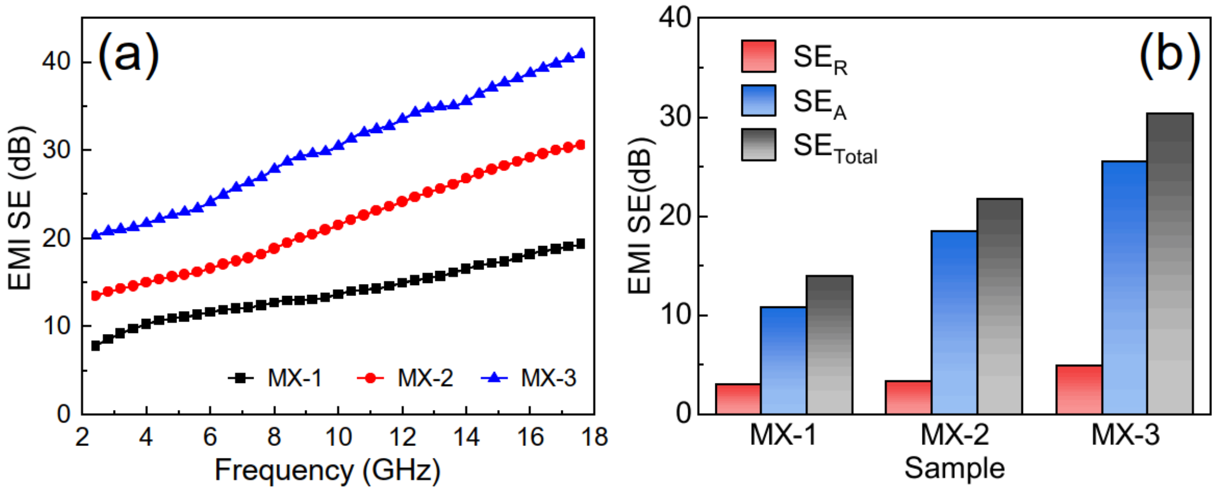

Figure S16. (a) The shielding performance of the sample MX-1, MX-2, and MX-3. (b) The corresponding average value of The total EMI shielding effectiveness (SE_{Total}), absorption efficiency (SE_A), and reflection efficiency (SE_R) of 2-18 GHz.

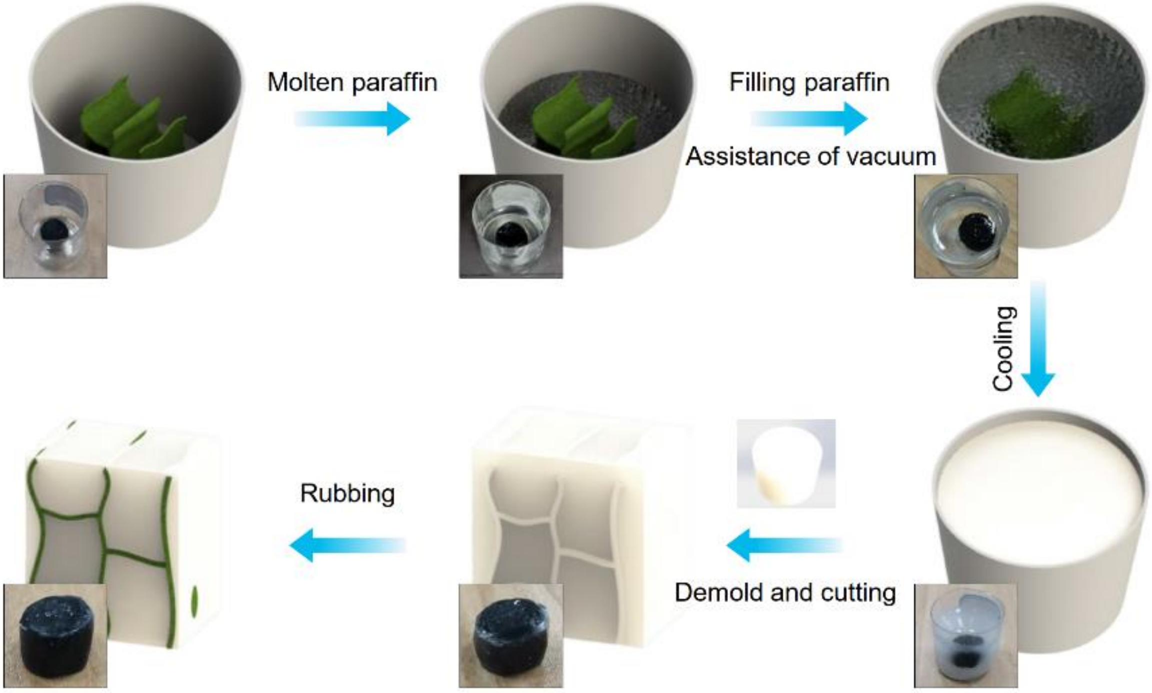

Figure S17. The process of the molten paraffin impregnated in the aerogel samples

Table S1. List of labels about all prepared aerogel samples.

Materials	Labels
MXene aerogel prepared with 10 mg/mL $Ti_3C_2T_x$ dispersion	MX-1
MXene aerogel prepared with 20 mg/mL $Ti_3C_2T_x$ dispersion	MX-2
MXene aerogel prepared with 30 mg/mL $Ti_3C_2T_x$ dispersion	MX-3
MXene aerogel prepared with 40 mg/mL $Ti_3C_2T_x$ dispersion	MX-4
MXene aerogel prepared with 50 mg/mL $Ti_3C_2T_x$ dispersion	MX-5
100 ALD MoS_2 cycles deposited on MX-3	MSX-1
200 ALD MoS_2 cycles deposited on MX-3	MSX-2
300 ALD MoS_2 cycles deposited on MX-3	MSX-3
400 ALD MoS_2 cycles deposited on MX-3	MSX-4
500 ALD MoS_2 cycles deposited on MX-3	MSX-5

Table S2. Microwave absorption performance of $Ti_3C_2T_x$-based aerogels

Materials	RL_{min} (dB)	EAB (GHz)	Thickness (mm)	Ref.
$Ti_3C_2T_x$/gelatin aerogel	-59.5	6.24	2	[13]
$Ti_3C_2T_x$@RGO aerogel	-31.2	5.4	2.05	[10]
$Ti_3C_2T_x$@GO aerogel microspheres	-49.1	2.9	1.2	[9]
$Ti_3C_2T_x$/PAA aerogel	-41.8	6.5(1.91mm)	4.5	[8]
TiO_2/$Ti_3C_2T_x$/RGO aerogel	−65.3	4.3	2.5	[14]
$Ti_3C_2T_x$/cellulose aerogel	-43.4	4.5	2	[15]
MoS_2/$Ti_3C_2T_x$ aerogel	-61.65	5.9(2mm)	4.53	this work

Table S3. The filling ratio of samples with different ALD cycles.

Samples	Weight of aerogel (g)	Weight of composite (g)	The filling ratio (wt%)	The average filling ratio (wt%)
MSX-1	0.0506	1.6891	97.004	96.828±0.209
	0.0359	1.0551	96.597	
	0.0405	1.2986	96.881	
MSX-2	0.0335	0.9743	96.562	96.43±0.183
	0.0375	0.9924	96.221	
	0.0343	0.9818	96.506	
MSX-3	0.052	1.3875	96.252	96.104±0.224
	0.0436	1.1512	96.213	
	0.0485	1.1675	95.846	
MSX-4	0.0548	1.2818	95.725	95.771±0.111
	0.0385	0.9385	95.898	
	0.0612	1.4202	95.691	
MSX-5	0.0346	0.7809	95.569	95.4±0.191
	0.0582	1.2106	95.192	
	0.0541	1.1862	95.439	

References

[1] X. L. Li, X. W. Yin, H. L. Xu, M. K. Han, M. H. Li, S. Liang, L. F. Cheng, L. T. Zhang, *ACS Appl. Mater. Interfaces* **2018**, *10*, 34524.

[2] J. Lu, Y. Li, W. Song, M. D. Losego, R. Monikandan, K. I. Jacob, R. Xiao, *ACS Nano* **2020**.

[3] J. Wang, L. Liu, S. Jiao, K. Ma, J. Lv, J. Yang, *Adv. Funct. Mater.* **2020**, *30*, 2002595.

[4] F. Pan, L. Z. Yu, Z. Xiang, Z. C. Liu, B. W. Deng, E. B. Cui, Z. Shi, X. Li, W. Lu, *Carbon* **2021**, *172*, 506.

[5] X. Li, X. Yin, C. Song, M. Han, H. Xu, W. Duan, L. Cheng, L. Zhang, *Adv. Funct. Mater.* **2018**, *28*, 1803938.

[6] L. W. Yan, C. Q. Hong, B. Q. Sun, G. D. Zhao, Y. H. Cheng, S. Dong, D. Y. Zhang, X. H. Zhang, *ACS Appl. Mater. Interfaces* **2017**, *9*, 6320.

[7] L. Y. Liang, G. J. Han, Y. Li, B. Zhao, B. Zhou, Y. Z. Feng, J. M. Ma, Y. M. Wang, R. Zhang, C. T. Liu, *ACS Appl. Mater. Interfaces* **2019**, *11*, 25399.

[8] Y. Dai, X. Y. Wu, Z. S. Liu, H. B. Zhang, Z. Z. Yu, *Compos. Pt. B-Eng.* **2020**, *200*, 9108263.

[9] Y. Li, F. B. Meng, Y. Mei, H. G. Wang, Y. F. Guo, Y. Wang, F. X. Peng, F. Huang, Z. W. Zhou, *Chem. Eng. J.* **2020**, *391*, 123512.

[10] L. B. Wang, H. Liu, X. L. Lv, G. Z. Cui, G. X. Gu, *J. Alloy. Compd.* **2020**, *828*, 10154251.

[11] Y. Li, F. B. Meng, Y. Mei, H. G. Wang, Y. F. Guo, Y. Wang, F. X. Peng, F. Huang, Z. W. Zhou, *Chem. Eng. J.* **2020**, *391*, 123512.

[12] T. Li, D. Zhi, Y. Chen, B. Li, Z. Zhou, F. Meng, *Nano Res.* **2020**, *13*, 477.

[13] M. L. Yang, Y. Yuan, Y. Li, X. X. Sun, S. S. Wang, L. Liang, Y. H. Ning, J. J. Li, W. L. Yin, Y. B. Li, *ACS Appl. Mater. Interfaces* **2020**, *12*, 33128.

[14] Y. Tong, M. He, Y. M. Zhou, S. X. Nie, X. Zhong, L. D. Fan, T. Y. Huang, Q. Liao, Y. J. Wang, *ACS Sustain. Chem. Eng.* **2018**, *6*, 8212.

[15] Y. Jiang, X. Xie, Y. Chen, Y. J. Liu, R. Yang, G. X. Sui, *J. Mater. Chem. C* **2018**, *6*, 8679.
